# Sex differences in patients’ recovery following an acute Achilles tendon rupture – a large cohort study

**DOI:** 10.1186/s12891-022-05875-9

**Published:** 2022-10-13

**Authors:** Elin Larsson, Annelie Brorsson, Malin Carling, Christer Johansson, Michael R Carmont, Katarina Nilsson Helander

**Affiliations:** 1 The Department of Orthopaedics, Sahlgrenska University Hospital Mölndal, Institute of Clinical Sciences at Sahlgrenska Academy, Gothenburg University, Gothenburg , Sweden; 2 IFK Kliniken Rehab, Gothenburg, Sweden; 3 The Department of Orthopaedics, Institute of Clinical Science at Sahlgrenska Academy, Gothenburg University, Gothenburg, Sweden; 4 The Department of Trauma & Orthopaedic Surgery, Princess Royal Hospital, Shrewsbury & Telford Hospital NHS Trust, Shropshire, UK

**Keywords:** Achilles tendon rupture, ATR, Sexes, Treatment, Achilles tendon total rupture score

## Abstract

**Introduction:**

The incidence of Achilles tendon ruptures (ATR) has increased over the past few decades. Treatment may be individualised based upon multiple factors including age, pre-injury activity level and the separation of the ruptured tendon ends. Several studies indicate that women may have a poorer self-reported and clinical outcome compared with men, but the number of women in these studies is often small due to the different incidence of ATR between the genders.

**Aims:**

The primary aim of this study was to evaluate whether there is a difference in self-reported outcome after an acute ATR between women and men at one to five years following injury. The second aim was to compare the outcome between the surgically and non-surgically treated patients.

**Methods:**

Data were obtained from the medical charts of patients treated for an acute ATR between 1 and 2015 and 31 December 2020 at Sahlgrenska University Hospital/Mölndal. The Achilles tendon total rupture score (ATRS) and additional questions relating to treatment and recovery were determined. A multiple regression analysis was performed to isolate the impact of sex when comparing the patient-reported outcome between women and men.

**Results:**

A total of 856 patients were included of which 66% participated prospectively. Sex, BMI and age were found to be significant factors influencing the total ATRS score. Female gender resulted in a lower ATRS, 7.8 points (CI = 3.3 to 12.3), than male gender. It was found that treatment did not significantly predict the results of the ATRS.

**Conclusion:**

To our knowledge, this is the first report with a larger number of women included showing that female sex predicts inferior self-reported results after an acute ATR.

## Background

An acute Achilles tendon rupture (ATR) is a common injury with an increasing incidence, particularly in the elderly population [1]. Recent research has suggested that this is a result of ageing adults participating in recreational sports [2]. A larger proportion of patients sustaining an ATR are men compared with women. The incidence has been reported as up to 55.2 per 100,000 person-years for men compared with 14.7 per 100,000 person-years for women [2].

The question of whether to treat an acute ATR surgically or non-surgically is the subject of a continuous debate within the orthopaedic community [3–5]. Studies have reported inconsistent results, leading to several paradigm shifts in primary treatment decisions over the last few decades [4, 5]. Myhrvold et al. [6] recently reported that surgery did not provide better self-reported outcome compared to non-surgical treatment following a randomized controlled trial. Even though there is a lack of consensus on how best to treat ATR patients, there are advantages and disadvantages to both treatment options. Meta-analyses report that better calf strength and a reduced re-rupture rate occur with surgical management [7, 8]. Surgical treatment, on the other hand, is reported significantly to increase the risk of complications, such as infection, wound problems and iatrogenic nerve injury [7].

Previous studies emphasised that women, compared with men, have inferior results and report poorer self-reported outcome following an ATR [9, 10]. Silbernagel et al. [9] reported that men had a greater improvement in heel-rise height (HRH) at 12 months after an ATR compared with women. Moreover, the authors revealed that surgically treated women reported a significantly poorer outcome when evaluated using the Achilles tendon total rupture score (ATRS) compared with men [9]. Saxena et al. [10] reported that women experienced a significant delay in return to activity compared with men, despite similar treatment protocols. Additionally, Vosseller et al. [11] concluded that women are less likely to suffer from an acute ATR but those women who do have greater predisposing comorbidity. Conversely, Bostick et al. [12] reported that being a woman predicted superior results in recovery and calf muscle endurance one year after a surgically treated ATR.

Although many studies have examined the self-reported and clinical outcome after an acute ATR, there is still a need for stronger evidence to confirm these findings regarding both treatments and differences between the sexes. The primary aim of this study was to evaluate whether there is a difference in self-reported outcome after an acute ATR one to five years after the injury, between women and men. Secondly, we aimed to compare the outcome between the surgical and non-surgical treatment group.

## Materials and methods

This was a retrospectively designed cohort study using prospectively acquired data, performed at the Department of Orthopaedics at Sahlgrenska University Hospital (SU), Mölndal, Sweden.

### Patient collection

All the patients that were registered with the ICD-10 code for an acute ATR (S86.0) or the ICD-10 code for a spontaneous rupture of a flexor tendon (M66.3) within the time period 1 January 2015 to 31 December 2020 at the emergency department at SU/Mölndal were identified.

### Data collection

A manual data collection was performed from patient medical charts; sex, age, injury level, date of injury, date of treatment and surgical or non-surgical treatment were documented. Both treatment groups received cast and brace with wedges, for the surgical group open repair was performed. For each patient, the diagnosis of an acute ATR was verified. The injury was considered acute if the day of injury was within 14 days of presentation. Patients were excluded if there was inadequate documentation, patients were lost to follow-up, were deceased, or had no valid address.

### Follow-up section

Patients were contacted by mail, one to six years following their initial injury, and invited to participate by completing a questionnaire. This consisted of the ATRS and further questions relating to treatment and recovery. These were (translated from Swedish to English): *“How satisfied are you with the treatment of your Achilles tendon rupture?”* and *“How would you describe your recovery after your Achilles tendon rupture as a percentage from 0-100? 0 stands for not recovered and 100 for fully recovered”.*

The ATRS consists of 10 questions which refer to limitations or difficulties following an Achilles tendon rupture. A maximum score of 100 implies no limitations and a full recovery. The ATRS has been found to be a valid and reliable instrument for measuring the outcome related to symptoms and physical activity after an ATR [13]. The patients could fill out their answers either on paper or on an internet-based application.

### Ethics

All the patients that were included in the study provided informed consent for enrolment. Ethical approval was given by the Swedish Ethical Review Authority, dnr 2021 − 01779. All methods were performed in accordance with the relevant guidelines and regulations.

### Statistical methods

Descriptive analyses were presented as the means and standard deviations (SD) for normally distributed variables and the median with interquartile range (IQR) for other variables. Categorical variables are presented as the number and percentage. An analysis of differences between the sexes was performed with an independent t-test or a non-parametric Mann-Whitney test. Categorical variables were analysed with the chi-square test of association.

A multiple linear regression analysis was performed to evaluate the impact of sex when comparing the patient-reported outcome between women and men. The regression model was adjusted for age, BMI and treatment regimen. The assumptions of multiple linear regression were verified. The level of significance was set at p < 0.05 and all the tests were two-sided. The software used in this study was R, R Core Team (2021). R: A language and environment for statistical computing. R Foundation for Statistical Computing, Vienna, Austria, and JASP, JASP Team (2022). JASP (Version 0.16.2).

## Results

Of the 856 eligible patients, 564 patients answered the questionnaire, a response rate of 66%, consisting of 129 women and 435 men respondents (Table 1).

**Table 1:** Patient demographics presented by the mean (SD)

Variable	Total n = 564	Women n = 129	Men n = 435	p-value
Age	49 (15.2)	45 (14.5)	50 (15.2)	0.24
BMI	26.2 (3.9)	25.3 (4.5)	26.5 (3.6)	**< 0.001**
Treatment				0.26
Surgery n (%)	158 (28.0%)	40 (31.0%)	118 (27.1%)	
Non-surgery n (%)	406 (72.0%)	89 (69.0%)	317 (72.9%)	

n = Number of patients

The multiple linear regression analysis included sex, BMI, age and treatment (Table 2). Sex, BMI and age were found to be significant factors influencing the total ATRS (r^2^ = 0.06). Female sex resulted in an inferior ATRS, 7.8 (CI = 3.3 to 12.3) points lower ATRS. The degree of symptoms increased with an increase in BMI, one unit higher predicted a 1.0-point reduction in the total ATRS. Increasing age was found to increase the limitation.

**Table 2:** Multiple linear regression analysis of ATRS

Variable	Regression coefficient		Standard error	95% CI	***p***
Sex (men)		7.8	2.27	3.3 to 12.3	**< 0.001**
BMI		-1.0	0.25	-1.5 to -0.5	**< 0.001**
Age		-0.2	0.07	-0.3 to -0.1	**0.006**
Treatment (non-surgery)		-1.1	2.13	-5.3 to 3.1	0.613

Women report a significantly poorer recovery expressed in per cent from 0 to 100. Moreover, women report inferior results in questions relating to satisfaction and recovery compared with men (Table 3).

**Table 3:** Patient-reported outcome one to five years after ATR

Variable	Total^*1*^ n = 564	Women^*1*^ n = 129	Men^*1*^ n = 435	p-value^*2*^
ATRS	82 (64,94)	79 (59,91)	84 (67,95)	**0.004**
Recovery in per cent	88 (75,95)	85 (75,90)	90 (75,95)	**0.005**
Satisfaction n (%)				**0.018**
Completely satisfied	256 (46.1%)	45 (35.4%)	211 (49.3%)	
Somewhat satisfied	182 (32.8%)	48 (37.8%)	134 (31.3%)	
Neither satisfied nor dissatisfied	72 (13.0%)	20 (15.7%)	52 (12.1%)	
Somewhat dissatisfied	27 (4.9%)	6 (4.7%)	21 (4.9%)	
Dissatisfied	18 (3.2%)	8 (6.3%)	10 (2.3%)	
Missing (n)	9	2	7	
Recovery n (%)				**0.031**
To a full extent	154 (27.7%)	28 (22.0%)	126 (29.4%)	
To a large extent	294 (53.0)	72 (56.7%)	222 (51.9%)	
Neither	40 (7.2%)	7 (5.5%)	33 (7.7%)	
To a small extent	65 (11.7%)	18 (14.2%)	46 (11.0%)	
Not at all	2 (0.4%)	2 (1.6%)	0 (0%)	
Missing (n)	9	2	7	
Activity now n (%)				0.24
Much more active	19 (3.4%)	3 (2.4%)	16 (3.7%)	
Somewhat more	47 (8.5%)	9 (7.1%)	38 (8.9%)	
Same	213 (38.4%	56 (44.1%)	157 (36.7%)	
Somewhat less	206 (37.1%)	39 (30.7%)	167 (39.0%)	
Much less active	70 (12.6%)	20 (15.7%)	50 (11.7%)	
Missing (n)	9	2	7	

^*1*^ Median (IQR) or frequency (%)

^*2*^ Wilcoxon rank sum test; Pearson’s chi-square test

## Discussion

The most important finding in this study is that women have significantly lower ATRS scores after an acute Achilles tendon rupture compared with men, even when adjusted for BMI, age and treatment. Treatment, either surgical or non-surgical, was not shown to predict ATRS.

The lower self-reported outcome in women is consistent with the results presented by Silbernagel et al. [9] in 2015 and also those by Aujla et al. [14] in 2017. Silbernagel and co-workers revealed that women (n = 30) reported a lower self-reported outcome, measured by the ATRS, after an acute ATR, with scores of 82 points for women compared with 92 points for men (p = 0.021). This effect, however, only occurred in patients treated with surgical management [5]. In Aujla et al’s. [14] study, women also reported a lower score on the ATRS after an acute ATR, compared with men. Thirty-seven women were included in this analysis, 236 overall, but the analysis was not stratified by treatment regimen, only by sex [14].

This study potentially comprises the largest number of women evaluated following an ATR and confirms the findings of earlier studies that women appear to experience a poorer outcome after an acute ATR. Treatment was not, however, shown to predict self-reported outcome. In their study, Silbernagel et al. [9] reported that the lack of a significant difference in patient-reported outcome between women and men in the non-surgical group may be explained by the small number of included patients sustaining an ATR [9].

Data collection in registry studies aims to collect national incidences of ATR rather than samples of patients. Non-registration and a lack of awareness are potential weaknesses of this method. Swennergren Hansen et al. [15] report data from the Danish Achilles tendon Database (DADB). Data from 296 men and 70 women were extracted. Women scored 22 points lower than men at 12 months following injury. This agrees with the sex difference in this series, although the difference is much greater, by 14 points. The time of the reported score following rupture may influence comparison with this study. DADB scores were reported at 12 months compared with our follow-up time of one to five years in this study. The longer follow-up time may have enabled improved rehabilitation and fewer limitations. Women in this study had a lower ATRS of 7.8 points. There is an unofficial assumption that a difference of 10 points is a clinically relevant difference [13]. It is worth noting that the confidence interval is fairly wide, ranging from 3.3 to 12.3, where the upper limit is beyond the 10-point clinical cut-off.

Cramer et al. [16] studied further data from the DADB, including 100 women and 416 men at baseline, four months, six months and 12 months following ATR. No significant difference in changes in ATRS were observed between the sexes [16]. These results, however, contrast with the findings from the present study. The fact that women report a lower ATRS score at baseline is a possible explanation for the non-significant difference between the sexes. It could therefore be hypothesised that women have a lower ATRS in general rather than a lower score at follow-up evaluation. The lack of a baseline ATRS in the present study needs to be considered in relation to this study’s findings. However, it must also be remembered that the ATRS has not been validated for evaluating “healthy” individuals at baseline pre-injury.

Further analyses of the questions relating to satisfaction with the treatment and recovery after the injury stratified by sex are consistent with the sex differences in the ATRS. These results confirm the association of inferior outcome among women after an ATR.

This study suggests that being a woman leads to a lower ATRS, but the reason for this is not known. It might be due to the design of the self-reported questions in the ATRS questionnaire not being completely addressed to women. Sex-differences in outcome have been identified in ankle fractures, another lower extremity injury. Balaji et al. [17] found that gender was a predictor of outcome after an unstable ankle fracture, with women having a poorer outcome [17] .

As mentioned in the background, several RCT:s have aimed to compare surgical and non-surgical treatment. Our results, showing no difference in self-reported outcome between surgical or non-surgical treatment are consistent with the most recent published [4, 6]. However, there is a large difference in size between the treatment groups with almost 70% treated non-surgical which is of importance to keep in mind while drawing conclusions.

There are limitations to this study. One is the low response rate which limits the conclusions that can be drawn from this work. It is not possible to determine the degree of satisfaction with recovery from injury for those that have not responded. Patients who responded could be either happy or unhappy with their treatment and so be motivated to respond. Other possible explanations for the low response rate are that patients may have changed address or that there was a language barrier since the questions were only in Swedish. A drop-out analysis was performed for non-responders. Out of the 564 patients eligible, there was a drop out of 292 patients. There was a greater proportion of males compared to females (22% females, 37% males, p < 0.001) of these patients and they were significantly younger (mean (SD)) than the respondent patients (42.5 (13.7), p < 0.001).

Another limitation of the study is the wide range of follow-up times between patients. Patients were contacted between January 2015 and December 2020, from 1.5 to 5.5 years following injury. This gives a range of four years for responses and patient function may change during this period. Olsson et al. [18], however, reported that there was no major improvement in the ATRS between the one- and two-year evaluations following rupture. Brorsson et al. [19] performed an evaluation seven years after injury on patients that suffered an acute ATR and it showed that there was no significant difference in scores on the ATRS between two years and seven years after the injury. Both studies were based on patients from previous randomised controlled trials [4, 20].

The main strength of this study is the large sample size (n = 564), especially women (n = 129). The large number of women make the results more robust and generalisable. Another strength is the fact that this retrospective study includes patients and evaluations at a single centre and so the completion of the ATRS will not suffer from centre-to-centre variation.

## Conclusion

The results based on this large cohort are broadly consistent with the majority of the studies reporting that being a woman can be regarded as a predictor of inferior results. Future research might apply research methods aiming to investigate the reason why women report inferior results compared with men. This study provides useful information when individualising treatment options for patients following an ATR.

## Data Availability

The datasets generated and analysed during the current study are not publicly available due to confidential information but are available from the corresponding author on reasonable request.

## Declarations

All patients that were included in the study provided informed consent for enrollment.
